# *CSF3R-*mutated chronic neutrophilic leukemia: long-term outcome in 19 consecutive patients and risk model for survival

**DOI:** 10.1038/s41408-018-0058-7

**Published:** 2018-02-15

**Authors:** Natasha Szuber, Christy M. Finke, Terra L. Lasho, Michelle A. Elliott, Curtis A. Hanson, Animesh Pardanani, Ayalew Tefferi

**Affiliations:** 1 Division of Hematology, Mayo Clinic, Rochester, MN USA; 2 Division of Hematopathology, Department of Laboratory Medicine, Mayo Clinic, Rochester, MN USA

Chronic neutrophilic leukemia (CNL) is a rare *BCR*-*ABL* negative myeloproliferative neoplasm (MPN), whose molecular pathogenesis transitioned from obscurity to the limelight with the seminal identification of oncogenic colony stimulating factor 3 receptor (*CSF3R*) mutations in the vast majority of CNL patients in 2013^1^. The unifying features of CNL consist of sustained mature neutrophil proliferation, bone marrow granulocytic hyperplasia, and hepatosplenomegaly, and while clinical manifestations and disease course remain heterogeneous, prognosis is often unfavorable^2^. Due to both the historically challenging diagnostic confirmation and the rarity of CNL, comprehensive analyses of World Health Organization (WHO)-defined, molecularly annotated populations of CNL patients have been scarce and often limited by small cohorts. Although the more common *CSF3R*T618I is perceived as the “hallmark” genetic lesion, there is little information on how individuals harboring *other CSF3R* mutations may be distinguished from their *CSF3R*T618I-mutated counterparts. Furthermore, while there is some evidence that variables such as high leukocyte count^3^, thrombocytopenia, and the presence *ASXL1* mutations may define a higher-risk subset of CNL patients^2^, there are limited data on prognostic factors and long-term survival in CNL and consequently, no existing operational risk model or prognostic scoring system. The objective of this study was to determine the characteristics, treatment patterns, and long-term overall survival (OS) in 19 consecutive WHO-defined *CSF3R*-mutated CNL patients—the largest cohort to date. Furthermore, we sought to identify and integrate adverse prognostic factors into a risk model predictive of inferior overall survival in CNL.

Nineteen consecutive patients evaluated at the Mayo Clinic harboring *CSF3R* mutations and meeting WHO criteria for CNL^4^ were retrospectively identified. Details of clinical characteristics, laboratory parameters including molecular and cytogenetics data, treatment regimens and responses, as well as disease evolution were carefully abstracted from medical records. Diagnosis was confirmed in all cases by review of peripheral blood counts and smears, and bone marrow (BM) aspirates and biopsies. Mutation analysis of *CSF3R*, *ASXL1*, and *SETBP1* was conducted as previously described^5, 6^. Analyses were based on clinical and laboratory parameters obtained at diagnosis. OS was calculated as an interval from the time of diagnosis to last follow-up or death. Survival analysis was performed by the Kaplan–Meier method and differences assessed using the log-rank test. Conventional statistical methods were used for all analyses. Statistical analyses were performed using Stat View software (SAS Institute, Cary, NC, USA).

From October 1995 to October 2017, 19 consecutive WHO-defined *CSF3R*-mutated patients were evaluated at the Mayo Clinic. Detailed clinical and laboratory characteristics of this cohort are presented in Table 1. Mutations in *CSF3R* included the classical T618I mutation in 14 patients and other mutations including M696T in 2 patients and T640N, c.2215C > T truncation mutation, and I598I SYN in one patient each. Median age was 68 (range 26–87 years) and there was a slight preponderance of males (58%). Overall, few patients had prior cytotoxic exposure or a family history of hematological neoplasm (20–21% each). When stratified according to the presence of *CSF3R*T618I versus other *CSF3R* mutations, those carrying T618I were older (median age 74 versus 59 years), showed greater male predominance (64 versus 40% male), had a more frequent history of thrombosis (54 versus 20%) and hemorrhage (39 versus 0%), and more commonly presented palpable splenomegaly (54 versus 40%) and an abnormal karyotype (14 versus 0%) at diagnosis, though these did not achieve statistical significance. Further, those harboring *CSF3R*T618I presented a trend towards higher leukocyte counts (76.4 versus 34 × 10^9^/L) and lower hemoglobin values (9.6 versus 13.6 g/dL) as well as significantly lower platelet counts (158 versus 299 × 10^9^/L; *p* = 0.04) compared to individuals carrying other *CSF3R* mutations. The mutational frequencies of *SETBP1* and *ASXL1* mutations respectively were: overall 32 and 47%; in *CSF3R*T618I-mutated individuals 36 and 43%; and in those with other *CSF3R* mutations 20 and 60%, though differences between the two groups were not statistically significant. Two of fourteen *CSF3R*T618I-mutated (14%) and one of five patients with other *CSF3R* mutations (20%) evolved to acute myeloid leukemia (AML) while one of fourteen (7%) and one of five (20%) respectively evolved to chronic myelomonocytic leukemia (CMML). Median time to last follow-up or death was 22.4 months and was significantly shorter in *CSF3R*T618I-mutated patients compared to those carrying other *CSF3R* mutations (17.2 versus 42.7 months; *p* = 0.03).

**Table 1:** Characteristics and outcomes in chronic neutrophilic leukemia patients with *CSF3R*T618I mutations versus other *CSF3R* variants (*n* = 19)

Characteristics	All patients	*CSF3R*T618I-mutated	Other *CSF3R* mutations^a^	*p*-value
	(*n* = 19)	(*n* = 14)	(*n* = 5)	
Median age at diagnosis (years)	68	74	59	0.09
Gender, male (%)	11 (58%)	9 (64%)	2 (40%)	0.35
Prior cytotoxic exposure	4/19 (21%)	3/14 (21%)	1/5 (20%)	0.95
Family history hematological neoplasm	4/19 (21%)	3/14 (21%)	1/5 (20%)	0.95
History of thrombosis	8/18 (44%)	7/13 (54%)	1/5 (20%)	0.20
History of hemorrhage	5/18 (28%)	5/13 (39%)	0/5 (0%)	0.10
Presence of palpable splenomegaly (%)	9/18 (50%)	7/13 (54%)	2/5 (40%)	0.60
Leukocyte count (x10^9^/L)	65.9	76.4	34.0	0.40
Leukocytes >60 × 10^9^/L^b^	10/19 (53%)	9/14 (64%)	1/5 (20%)	0.09
Hemoglobin (g/dL)	12.0	9.6	13.6	0.06
Hemoglobin <10 g/dL	9/19 (47%)	8/14 (57%)	1/5 (20%)	0.15
Platelet count (x10^9^/L)	227	158	299	**0.04**
Platelets <160 × 10^9^/L^b^	8/19 (42%)	8/14 (57%)	0/5 (0%)	**0.03**
Metamyelocytes + myelocytes (%)	2%	2%	2%	0.82
Monocytes (%)	2%	1%	5%	0.11
LDH (U/L)	234	234	234	0.50
Abnormal karyotype (%)	2/19 (11%)	2/14 (14%)	0/5 (0%)	0.37
*SETBP1* mutation (%)	6/19 (32%)	5/14 (36%)	1/5 (20%)	0.52
*ASXL1* mutation (%)	9/19 (47%)	6/14 (43%)	3/5 (60%)	0.51
Therapy regimens				
First line therapy				
Hydroxyurea	14/17 (82%)	9/12 (75%)	5/5 (100%)	NA
Interferon-alpha	1/17 (6%)	1/12 (8%)	–	
Other agent^c^	2/17 (12%)	2/12 (17%)	–	
Second line therapy				
Ruxolitinib	3/10 (30%)	2/8 (25%)	1/2 (50%)	
Stem cell transplant	1/10 (10%)	1/8 (13%)	–	
Other agent^c^	6/10 (60%)	5/8 (62%)	1/2 (50%)	
Third line therapy				
Stem cell transplant	1/6 (17%)	–	1/2 (50%)	
Ruxolitinib	1/6 (17%)	1/4 (25%)	–	
Other agent (Cladribine or Decitabine)	2/6 (33%)	1/4 (25%)	1/2 (50%)	
Combination therapy^d^	2/6 (33%)	2/4 (50%)	–	
Requiring 2nd line therapy	10 (53%)	8 (57%)	2 (40%)	0.51
Requiring 3rd line therapy or more	6 (32%)	4 (29%)	2 (40%)	0.64
Status last follow-up, dead (%)	10 (53%)	8 (57%)	2 (40%)	0.51
Evolution				
AML (%)	3/19 (16%)	2/14 (14%)	1/5 (20%)	0.76
CMML (%)	2/19 (11%)	1/14 (7%)	1/5 (20%)	0.42
Time to last follow-up or death	22.4 months	17.2 months	42.7 months	**0.03**

Bold indicate statistically significant values

*AML* acute myeloid leukemia, *CMML* chronic myelomonocytic leukemia, *LDH* lactate dehydrogenase, *NA* not available

^a^Other *CSF3R* mutations: M696T (*n* = 2), T640N, c.2215C>T truncation mutation, and I598I SYN (*n* = 1 each)

^b^Leukocyte count >60 and platelet count <160 × 10^9^/L cut-off values determined by ROC analysis

^c^Other therapy regimens: thalidomide, tyrosine kinase inhibitors (imatinib, dasatinib), cladribine, azacitidine, and hydroxyurea plus thalidomide

^d^Combination therapies included hydroxyurea plus nilotinib in one patient and splenectomy followed peri-operatively by hydroxyurea plus interferon-alpha in another

**Fig. 1:**
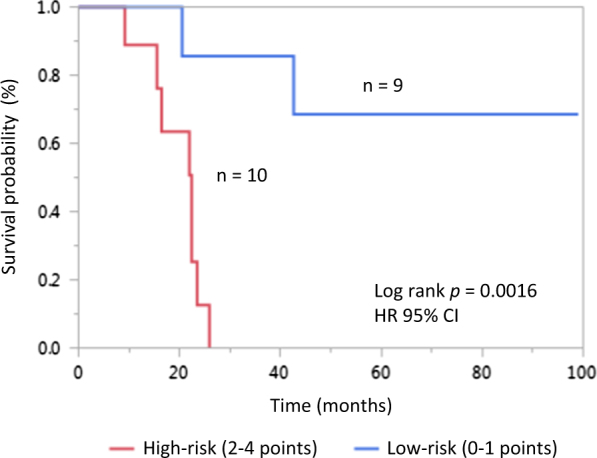
Risk-stratified Kaplan–Meier survival curves for 19 *CSF3R*-mutated CNL patients. Points were attributed for ROC analysis-defined cut-offs of platelet count <160 × 10^9^/L (2 points), leukocyte count >60 × 10^9^/L (1 point) and presence of *ASXL1* mutation (1 point) according to their respective risks ratios and patients stratified into low-risk (0–1 points) or high-risk (2–4 points) groups using this prognostic risk model (log rank *p* = 0.0016)

Using a receiver operating characteristics (ROC) analysis, optimal cut-off points for defining low/high-risk disease were determined for leukocyte and platelet counts. On univariate analysis, platelet count below 160 × 10^9^/L, leukocyte count above 60 × 10^9^/L, and presence of an *ASXL1* mutation were associated with significantly inferior OS (*p* < 0.05) and all three maintained significance on multivariate analysis with respective *p*-values of 0.001, 0.036, and 0.016. Based on these parameters, weighted risk points were attributed commensurate with the risk ratio (RR) of each variable to define a scoring system predictive of CNL patient survival: platelets below 160 × 10^9^/L (RR = 16; 2 points), leukocytes above 60 × 10^9^/L (RR = 5, 1 point), and presence of *ASXL1* mutation (RR = 6, 1 point). Using this risk model, patients were assigned a low-risk (0–1 points; *n* = 9) or high-risk (2–4 points; *n* = 10) designation and risk-stratified Kaplan–Meier survival curves confirmed significantly decreased OS in high-risk patients (median OS 22.4 months versus not yet reached; log rank *p* = 0.0016) (Fig. 1).

Treatment regimens and responses are presented in Table 1. The majority of patients were treated with hydroxyurea as a first-line agent (82%) and it was ultimately received by all (100%) at some point over their disease course. Most patients (53%) required second-line therapy and nearly a third (32%) required three lines of treatment or more. In this cohort, four patients, all having previously been exposed to hydroxyurea, received treatment with JAK inhibitor ruxolitinib. In three instances, ruxolitinib was administered as a second-line therapy and in one case, as a third-line agent. Reponses to ruxolitinib were varied: in one case, treatment was ongoing with favorable response but had been initiated recently (~2 months prior to data collection), in two other cases there was an initial response but eventual worsening of leukocytosis requiring subsequent additional therapies (duration of responses ~9.5 and 36 months, respectively), and in one case, ruxolitinib was received during blast phase disease as a “bridge” to transplant for a duration of only ~0.5 months; this patient ultimately had a favorable outcome and was alive at last follow-up, approximately 46 months from initial diagnosis. Two patients ultimately underwent hematopoietic stem cell transplant (HSCT), both 3 months following blast transformation; while one patient succumbed to complications of HSCT (veno-occlusive disease and ultimately death secondary to infection), the other (patient having received ruxolitinib pre-HSCT) experienced a favorable outcome and showed no signs of relapse ~40 months post-transplant.

As the largest-scale report of consecutive *CSF3R*-mutated CNL patients to date, this study yielded several novel and clinically useful findings. First, the assessment of long-term outcomes confirmed the generally aggressive course of CNL with a median survival of less than 2 years, consistent with historical data^7^, and a requirement for two or more lines of therapy in most patients. Although only ~16% of patients formally evolved to AML, most patients eventually experienced a worsening of leukocytosis and an increased requirement for transfusions, regardless of therapeutic agent, and blast transformation was eventually suspected in a number of additional patients, though unconfirmed.

Second, by subdividing *CSF3R*-mutated CNL patients into mutational subgroups, specifically T618I versus other *CSF3R* mutations, we identified two phenotypically and prognostically distinct subsets of CNL patients. *CSF3R*T618I-mutated individuals cluster with adverse clinical characteristics, represent a prognostically less favorable group overall and likely correspond to the definitive molecularly-defined CNL entity. Further studies explicitly appraising the less prevalent, diversely-composed molecular subset of “other” *CSF3R* mutations will be required to validate these findings. Interestingly, the question of whether these non-T618I *CSF3R* mutations should still be considered accurate molecular markers of CNL is complex, requires further investigation, and cannot be addressed within the confines of this communication.

Importantly, we integrated the three variables predictive of inferior survival on multivariate analysis weighted for risk to develop low-risk and high-risk patient categories and an operational risk model for survival in CNL (platelet count <160 × 10^9^/L = 2 points, leukocyte count >60 × 10^9^/L = 1 point, and presence of *ASXL1* mutation = 1 point; low-risk 0–1 points, high-risk 2–4 points). This is the first prognostic scoring system to be reported for risk assessment in CNL. From a practical standpoint, we propose that individuals classified as high-risk be counseled regarding the prognostic implications of their disease, undergo closer monitoring for signs heralding disease transformation and perhaps even be considered earlier on for more intensive therapeutic approaches such as hematopoietic stem cell transplant.

Finally, although interesting preliminary data exist^6, 8–10^, there has been limited clinical experience using ruxolitinib in patients with CNL. Ruxolitinib was received by four patients in our cohort with varying responses. In all cases, it was successful in initially controlling leukocyte count and in one instance of application pre-HSCT, its potential contribution to a favorable post-transplant outcome remains uncertain. However, in 50% of the cases, there was an eventual, albeit variably-timed loss of response, suggesting that ruxolitinib has limited if any disease-modifying effect. Thus, while it may be justified to maintain this agent in our arsenal of CNL-directed therapy, identifying effective treatments capable of inducing durable remission are clearly an unmet need in this disease.
